# Validation and Adaptation of the Prosocial and Antisocial Behavior in Sport Scale to the Spanish context of Physical Education

**DOI:** 10.3390/ijerph17020477

**Published:** 2020-01-11

**Authors:** Rubén Trigueros, Antonio Alias, Ana M. Gallardo, Marta García-Tascón, José M. Aguilar-Parra

**Affiliations:** 1 Department of Psychology, Hum-878 Research Team, Health Research Centre, University of Almería, 04120 Almería, Spain; 2 Department of Education, University of Almería, 04120 Almería, Spain; aag344@ual.es; 3 Faculty of Sports, Universidad Católica de Murcia, 30107 Guadalupe-Murcia, Spain; amgallardo@ucam.edu; 4 Faculty of Sports Science, Univeridad Pablo de Olavide, 41704 Seville, Spain; margata@upo.es

**Keywords:** social behaviour, students, validation, physical education

## Abstract

Physical education (PE), by its own characteristics, is a subject where social communication is especially promoted. However, it is necessary to have tools that evaluate the social behaviour of students during PE classes. For this reason, we propose to validate and adapt the Prosocial and Antisocial Behavior in Sport Scale to the Spanish context of PE classes. The study involved 1081 students aged 12 to 18 (M = 14.83; SD = 1.27). The psychometric properties of the Prosocial Behavior Scale were analyzed through several statistical analyses. The results of the confirmatory factorial analysis and the exploratory factorial analysis supported the internal structure of the questionnaire. In addition, the scale was invariant to gender. Cronbach’s alpha values were higher than 0.70 in the factors and sub-factors, finally showing adequate levels of temporal stability. Taking into account the results achieved in the present study, PE teachers have an effective tool to assess the social and antisocial behaviour of their students’ students during PE classes.

## 1. Introduction

In recent years, multiple studies have shown the influence of physical education classes on the emotional, physical, and psychological development of students [1,2,3]. For this reason, many researchers have focused on the social behavior of students during physical education classes, since these are key to understanding the involvement and participation of physical education students [4,5]. In this sense, the educational curriculum refers to the fact that one of the fundamental objectives of physical education classes is the participation of students with respect and tolerance in the different physical activities, accepting the established rules and resolving conflicts through dialogue and mediation [6]. However, despite this interest, there is still little scientific evidence to try to measure and analyze social behaviors in the Spanish context of physical education. Hence, the aim of this research is to validate and adapt the Prosocial and Antisocial Behavior in Sport Scale (PABSS) by Kavussanu and Boardley [7] to the Spanish context of PE.

Social behaviors can be both positive and negative, being referred to in the form of prosocial and antisocial behaviors, these behaviors are linked to the two dimensions of morality [8,9]. Prosocial behaviour has been defined as a set of voluntary behaviours with the aim of establishing positive socially responsible, empathetic and cooperative relationships in order to benefit people [10], an example would be the student helping a partner in difficulty. On the contrary, antisocial behaviour has been defined as the set of behaviours destined to harm or despise others, thus establishing a negative relationship [11]. An example would be that student who insults a partner when he does something that is wrong.

Studies to date have traditionally focused on adolescent antisocial behaviors [12,13,14]. These studies have shown that antisocial behaviour negatively affects the adolescent’s temperament, cognitive capacity and relationship with peers, dramatically affecting their ability to solve problems and adapt socially [11]. In the educational field, antisocial behavior has been negatively related to motivation to study [15] and academic performance [16], and positively to frustration of psychological needs, depression, anxiety, and stress [17,18]. However, studies are now emerging that have focused on prosocial behavior, as it has an inhibitory effect on maladaptive social styles (e.g., aggressiveness, insult, and social shyness) [19,20]. In this sense, prosocial behavior is related to the development of positive interpersonal relationships, based on the acceptance of others (e.g., parents, siblings, teachers and peers) [21]. In addition, prosocial behavior is closely related to academic motivation [22], academic achievement [23], and satisfaction of psychological needs [24]. In this way, prosocial behavior can be considered a key factor in promoting social and academic competence of adolescents [25].

Among the tools developed so far to evaluate prosocial and antisocial behavior, the teenage inventory of social skills (TISS; Inderbitzen and Foster [26]) stands out. This scale consists of 40 items that measure prosocial behavior and aggressive behavior. However, this scale only focuses on behaviours linked to the day-to-day behaviour of adolescents and ignoring certain aggressive behavioural elements such as insults, threats and insults that are more present in adolescent behaviour than physical aggressions [27]. Therefore, Kavussanu and Boardley [7] developed the PABSS for the sporting context in order to have an effective tool with which to measure the social behaviour of adolescents. This scale was originally developed with 30 items which evaluate the pro-social and antisocial behaviors of athletes. Both factors are composed of two sub-factors called prosocial behaviors towards teammates and opponents, and antisocial behaviors towards teammates and opponents. To validate the questionnaires, they used a sample of 1213 athletes from various disciplines with an average age of 21.97 years. To validate the scale the authors performed a reliability analysis that revealed that each of the factors and sub-factors were above 0.70. In addition, the authors analyzed the factor structure of the scale through exploratory and confirmatory factor analysis. The results of the exploratory factorial analysis showed that the primary loads of some of the items with respect to their factor was less than 0.30, so they were eliminated, resulting in a final questionnaire of 20 items. Subsequently, the confirmatory factor analysis revealed acceptable adjustment indices for the 20 items. Finally, the gender invariance analysis revealed that the questionnaire was understood in a similar way by athletes.

Based on these antecedents, the aim of this research was to analyze the psychometric properties of the PABSS in order to validate and adapt it to the context of PE, especially in reference to those sports activities typical of secondary education. It is expected that the EFA and CFA of the proposed instrument (prosocial and antisocial behaviour scale of PE students) will offer adequate adjustment indices and will be invariant with respect to gender. In addition, internal consistency and temporal stability are expected to be adequate.

## 2. Method

### 2.1. Participants

**Figure 1:**
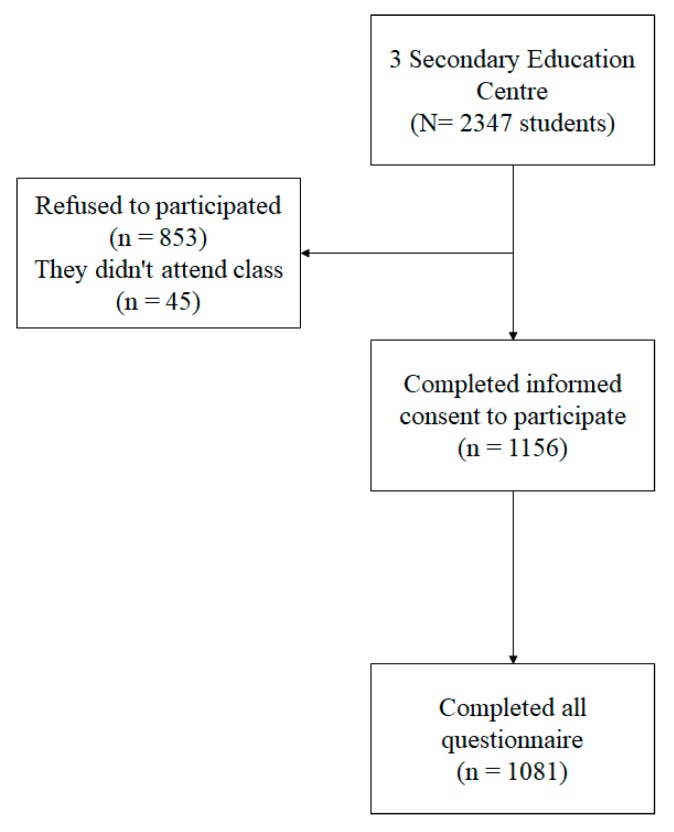
Sample flowchart.

The sample of the present study was composed of high school students belonging to the province of Almeria (Spain), being 512 boys and 569 girls. The average age of the students was 14.83 (SD = 1.27), ranging from 12 to 18 years (see Figure 1).

As for the exploratory factorial analysis, an independent sample of the previously described was used, consisting of 387 boys and 355 girls. The average age of the students was 14.52 (SD = 1.84), ranging from 12–18 years.

For the analysis of temporal stability, an independent sample was used, composed of 174 boys and 126 girls. The age of the students ranged from 12 to 18 years, with an average age of 14.27 (SD = 1.48).

The selection of the sample was non-probabilistic and incidental, since it was made according to those secondary schools that accepted to participate in the study. On the other hand, the criteria for participating in the study was the provision of informed consent by parents or legal guardians.

### 2.2. Instruments

To measure the prosocial and antisocial behaviors of PE students, the PABSS by Kavussanu and Boardley [7] was adapted and validated. The scale is made up of 20 items that are divided between four sub-factors that in turn are divided between two factors: pro-social behavior (towards teammates, towards opponents) and antisocial behavior (towards teammates, towards opponents). Items are answered on a Likert scale of 1 (Strongly disagree) to 7 (Strongly agree).

### 2.3. Procedure

The strategy used to fulfill the objective of validating the questionnaire to the Spanish context was the Hambleton [28] procedure. This procedure consists of the translation of the original questionnaire into Spanish by a group of translators with extensive experience in studies related to PE classes, and later, another group of translators translated the questionnaire from Spanish into the original language of the scale. The quality of the translation was considered on the basis of the goodness of coincidence with respect to the original version. Once the scale was obtained, it was analysed by three expert researchers in the field of PE with extensive experience, in such a way as to guarantee that the scale was well designed to measure the variables to be measured without losing the meaning of the original scale.

The scale obtained from the previous process was shown to the heads of several secondary educational centres in one of province of Andalusia, who were asked for their collaboration and informed of the aims pursued in the study. Before administering the questionnaires to PE students, they were required to give informed consent from their parents or legal guardians, and were subsequently informed of the aim of the study. A member of the research group was present while the students completed each of the questionnaires in case any of them had any questions. The students filled out all the questionnaires in about 15 min.

### 2.4. Data Analysis

The psychometric properties of the PABSS scale were examined to determine its validity and reliability in the Spanish PE context. First, an exploratory factorial analysis (EFA) was performed. Second, a confirmatory factorial analysis (CFA) was performed to test the factorial structure. Third, in order to analyze the invariance of the questionnaire, a multi-group analysis was carried out with respect to gender. In addition, various descriptive statistical analyses, an analysis of internal consistency across the Cronbach alpha, and an analysis of temporal stability were performed. The statistical packages SPSS 25 and AMOS 20 (IBM, Armonk, NY, USA) were used for the various analyses.

The maximum likelihood estimation method was used in the AFC together with the bootstrapping procedure since the Mardia coefficient was high (187.23). The estimators were considered robust as they were not affected by the lack of normality [29]. In order to be able to accept or reject the model tested, a set of adjustment indices has been taken into consideration [30]: χ^2^/df, acceptable values are considered those lower than 3; the comparative fit index, Tucker-Lewis index and incremental fit index incremental values are considered a good adjustment equal to or higher than 0.95; while the error indices Root mean square error of approximation plus its 90% confidence interval (CI), and standardized root mean square residual, are considered acceptable with values lower than or equal to 0.06.

## 3. Results

### 3.1. Exploratory Factorial Analysis

As can be seen in Table 1 the correlations between each item with respect to the total score of the scale that is in the general range between 0.79 and 0.87. In addition, the correlation between test items is higher than the cut-off point established at 0.30 [31] so all items must be maintained. In addition, exploratory factor analysis supports the existence of two factors, showing a saturation factor ranging from 0.76 to 0.85 for prosocial behaviors and from 0.75 to 0.83 for antisocials behaviors.

### 3.2. Confirmatory Factorial Analysis

**Figure 2:**
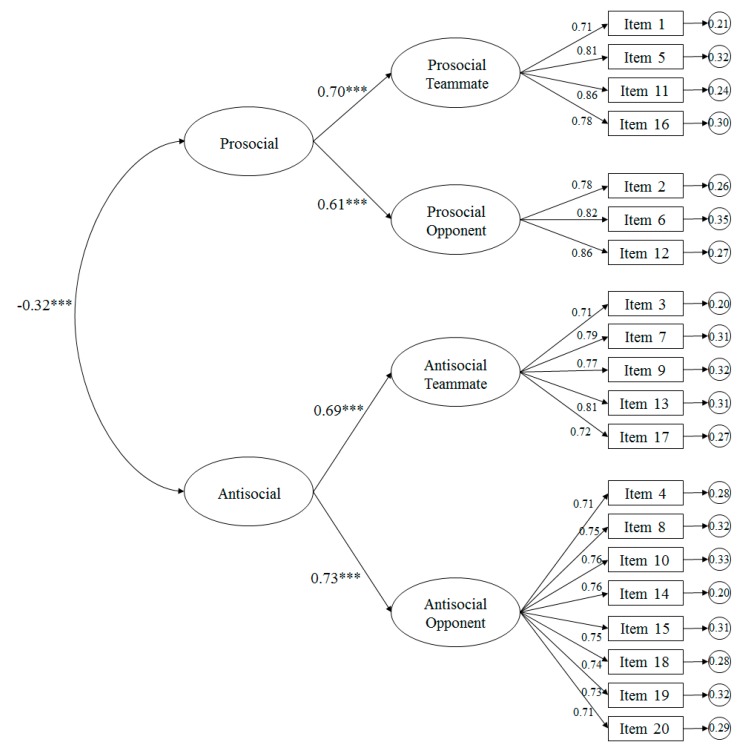
Diagram of the CFA belonging to the prosocial and antisocial scale in the context of the Physical Education. Note: *** *p*< 0.001.

The fit indices were adequate for the model tested (Figure 2): χ^2^ (165. N = 1081) = 456.89, *p* < 0.001; χ^2^/df = 2.77; CFI = 0.96; TLI = 0.96; IFI = 0.96; RMSEA = 0.062 (90% CI = 0.057–0.068); SRMR = 0.042. Standardized regression weights were statistically significant (*p* < 0.001), which ranged from 0.71 to 0.86. In addition, the factors correlated negatively, being –0.32 and statistically significant (*p* < 0.001).

### 3.3. Analysis of Invariance by Gender

In Table 2, the results of the multigroup analysis are presented in order to check whether the factor structure of the questionnaire is invariant with respect to gender. Table 2 shows the absence of significant differences between the unrestricted model (model 1) and the invariance model in measurement weights (model 2). However, there were significant differences between model 1 and the invariant structural covariance models (model 3) and the invariant measurement residue model (model 4).

To accept that the model is gender invariant, the minimum criterion is the absence of significant differences between model 1 and model 2 [32].

### 3.4. Descriptive Statistics, Correlation and Reliability Analysis

Table 3 displays how the two factors correlate negatively, revealing the non-reciprocity between the factors and sub-factors. In addition, Table 3 shows evidence of the reliability of the factors and sub-factors, the scores being above 0.70 [33]. 

Intraclass correlation coefficients (ICC) and their confidence intervals (CI) were calculated to analyse the temporal stability of the scale. The scores were: 0.82 (CI = 0.81–0.85) for prosocial behavior and 0.88 (CI = 0.84–0.92) for antisocial behavior.

## 4. Discussion

The aim of this study was to adapt and validate the PABSS to the Spanish context of PE classes in secondary school students. Thus, the present study has evaluated the psychometric properties of the instrument in a new scale, called prosocial and antisocial behaviour scale of PE students (PABS-PE; Appendix A), analysing its factorial structure and reliability in such a way as to demonstrate that the scale is made up of two factors.

Like the original scale, the results obtained in the EFA have shown the existence of two factors. On the other hand, the results obtained through the EFA support the factorial structure of four sub-factors and two factors being the negative correlation between them, these results are similar to those of the original scale [7]. As for the multigroup analysis, it showed that the factor structure of the PABS-PE was invariant with respect to gender given the existence of significant differences between model 1 and model 3. Therefore, the instrument can be used by both boys and girls when the scale is understood in a similar way by both groups, analyzing in this way the possible differences between both populations.

As for the internal consistency analyses, they revealed scores above 0.80, which are higher than those of the original questionnaire [7]. In addition, the analysis of temporal stability revealed that the scale was understood by the population in a similar way after a period of time. Likewise, the bivariate correlations between the factors and sub-factors showed the same polarity as the EFA and were lower than 0.85, thus suggesting that the scale has an adequate level of discriminant validity.

Taking into account the results achieved in this study, PE teachers have an effective tool with which to assess the prosocial and antisocial behaviour of their students. Teachers must implement educational programs that model caring and respectful behavior to develop students’ social skills and their connection to the school community to promote their prosocial behavior [34]. 

Although a valid and reliable instrument has been developed to measure prosocial and antisocial behaviour in PE, the study has some limitations that should be noted. First, the scale was constructed on the basis of data from a given socio-cultural context. Secondly, the scale has only taken into account secondary school students and no other ages. In addition, future research should further develop the current scale by distinguishing other dimensions of pro-social and anti-social behaviour directed towards classmates.

In conclusion, the results of the present study support the usefulness of PABS-PE, as an instrument that shows evidence of reliability and validity for measuring the pro-social and antisocial behaviour of PE students. On the other hand, the scale has an adequate factorial structure and is understood in a similar way by both boys and girls. In this way, this instrument can contribute significantly to a better understanding of the behavioural processes of students in relation to their classmates during PE classes.

## Figures and Tables

**Table 1:** Correlations between each item and the total scale score.

Items	Correlations Item-Test	Saturation Factor of Each Item with Its Factor
1	0.80 **	0.77 F1
2	0.82 **	0.77 F1
3	0.83 **	0.82 F2
4	0.81 **	0.81 F2
5	0.87 **	0.83 F1
6	0.86 **	0.82 F1
7	0.85 **	0.80 F2
8	0.83 **	0.86 F2
9	0.80 **	0.83 F2
10	0.81 **	0.76 F2
11	0.85 **	0.77 F1
12	0.89 **	0.82 F1
13	0.82 **	0.83 F2
14	0.83 **	0.82 F2
15	0.80 **	0.78 F2
16	0.84 **	0.84 F1
17	0.83 **	0.81 F2
18	0.82 **	0.75 F2
19	0.79 **	0.78 F2
20	0.80 **	0.76 F2

Note: F1 = Prosocial Behavior; F2 = Antisocial Behavior. ** *p* < 0.01.

**Table 2:** Gender Invariance Analysis.

Two Factors Model										
Models	χ^2^	*df*	χ^2^/*df*	Δχ^2^	Δ*df*	CFI	TLI	IFI	RMSEA (IC 90%)	SRMR
Model 1	951,91	330	2.88	-	-	0.96	0.95	0.96	0.055 (0.046–0.063)	0.044
Model 2	1014.21	346	2.93	16.92	16	0.96	0.95	0.96	0.054 (0.045–0.063)	0.046
Model 3	1095.47	348	3.15	19.29	18	0.96	0.95	0.96	0.053 (0.045–0.063)	0.047
Model 4	1112.01	351	3.17	28.34 *	21	0.96	0.95	0.96	0.053 (0.045–0.062)	0.047
Model 5	1144.33	355	3.22	39.88 ***	25	0.95	0.95	0.95	0.051 (0.043–0.061)	0.048
Model 6	1227.55	375	3.27	87.82 **	45	0.95	0.95	0.95	0.052 (0.041–0.064)	0.051

* *p* < 0.05; ** *p* < 0.01; *** *p* < 0.01. Note: Comparative Fit Index (CFI), Tucker-Lewis Index (TLI), Incremental Fit Index (IFI), Root Mean Square Error of Approximation (RMSEA), Standardized Root Mean Square Residual (SRMR).

**Table 3:** Descriptive statistics and bivariate correlations.

	*M*	*SD*	*α*	Range	1	1.1	1.2	2	2.1	2.2
1. Prosocial	4.67	0.89	0.86	1–7		0.48 ***	0.38 **	−0.32 ***	−0.48 ***	−0.35 ***
1.1. Prosocial Teammate	5.18	1.10	0.83	1–7			0.51 ***	−0.34 ***	−0.41 ***	−0.39 ***
1.2. Prosocial Opponent	4.10	0.67	0.80	1–7				−0.41 ***	−0.55 ***	−0.30 ***
2. Antisocial	2.34	0.72	0.87	1–7					0.52 ***	0.61 ***
2.1. Antisocial Teammate	1.59	0.69	0.81	1–7						0.44 ***
2.2. Antisocial Opponent	2.67	1.22	0.79	1–7						

*Note:* *** *p*< 0.001; ** *p* < 0.01.

## Appendix A

The questionnaire was validated in Spanish.

1. Animó a mis compañeros de equipo

2. Felicitó a mis compañeros de equipo por hacerlo bien

3. Trato de ayudar positivamente a mis compañeros de equipo

4. Trato de ayudar constructivamente a mis compañeros de equipo

5. Ayudó a los oponentes lesionados

6. Pido que se interrumpa el juego cuando un adversario se lesiona

7. Ayudó a los oponentes a levantarse del suelo

8. Amenazo verbalmente a mis compañeros de equipo

9. Insulto a mis compañeros de equipo

10. Discuto con mis compañeros de equipo

11. Criticó a mis compañeros de equipo

12. Muestro mi frustración por el mal juego de mis compañeros de equipo

13. Trató de herir a mis oponentes

14. Trató de lesionar a mis oponentes

15. Cometo faltas deliberadamente

16. Distraigo intencionalmente a mis oponentes

17. Me suelo vengar después de una falta grave

18. Rompo intencionalmente las reglas del juego

19. Intimido físicamente a mis oponentes

20. Critico a mis oponentes
